# Biomechanical Properties of Paraspinal Muscles Influence Spinal Loading—A Musculoskeletal Simulation Study

**DOI:** 10.3389/fbioe.2022.852201

**Published:** 2022-06-02

**Authors:** Masoud Malakoutian, C. Antonio Sanchez, Stephen H. M. Brown, John Street, Sidney Fels, Thomas R. Oxland

**Affiliations:** ^1^ Department of Mechanical Engineering, University of British Columbia, Vancouver, BC, Canada; ^2^ ICORD, University of British Columbia, Vancouver, BC, Canada; ^3^ Department of Electrical and Computer Engineering, University of British Columbia, Vancouver, BC, Canada; ^4^ Department of Human Health and Nutritional Sciences, University of Guelph, Guelph, ON, Canada; ^5^ Department of Orthopaedics, University of British Columbia, Vancouver, BC, Canada

**Keywords:** musculoskeletal model, lumbar spine, biomechanics, intradiscal pressure, muscle, sarcomere, passive stiffness

## Abstract

Paraspinal muscles are vital to the functioning of the spine. Changes in muscle physiological cross-sectional area significantly affect spinal loading, but the importance of other muscle biomechanical properties remains unclear. This study explored the changes in spinal loading due to variation in five muscle biomechanical properties: passive stiffness, slack sarcomere length (SSL), *in situ* sarcomere length, specific tension, and pennation angle. An enhanced version of a musculoskeletal simulation model of the thoracolumbar spine with 210 muscle fascicles was used for this study and its predictions were validated for several tasks and multiple postures. Ranges of physiologically realistic values were selected for all five muscle parameters and their influence on L4-L5 intradiscal pressure (IDP) was investigated in standing and 36° flexion. We observed large changes in IDP due to changes in passive stiffness, SSL, *in situ* sarcomere length, and specific tension, often with interesting interplays between the parameters. For example, for upright standing, a change in stiffness value from one tenth to 10 times the baseline value increased the IDP only by 91% for the baseline model but by 945% when SSL was 0.4 μm shorter. Shorter SSL values and higher stiffnesses led to the largest increases in IDP. More changes were evident in flexion, as sarcomere lengths were longer in that posture and thus the passive curve is more influential. Our results highlight the importance of the muscle force-length curve and the parameters associated with it and motivate further experimental studies on *in vivo* measurement of those properties.

## 1 Introduction

Paraspinal muscles are vital to the functionality of the spine and their dysfunction is deemed a major risk factor for a variety of spinal disorders including spinal deformity (Sinaki et al., 1996; Roghani et al., 2017), adjacent segment disease (Kim et al., 2016; Malakoutian et al., 2016a), and lower back pain (Nicolaisen and Jorgensen, 1985; Mannion, 1999; Goubert et al., 2017; Masaki et al., 2017). To what extent muscle dysfunction is involved in the development of these conditions is unknown. Musculoskeletal models of the spine provide estimates of muscle and spinal loading for various conditions and daily activities and thus provide unique insight into the biomechanical performance of the system since direct measurement of *in vivo* spinal forces and moments is not feasible with current technology. This knowledge could enhance our understanding of the etiology of spinal conditions and help in the development of better treatment or prevention strategies.

**FIGURE 1:**
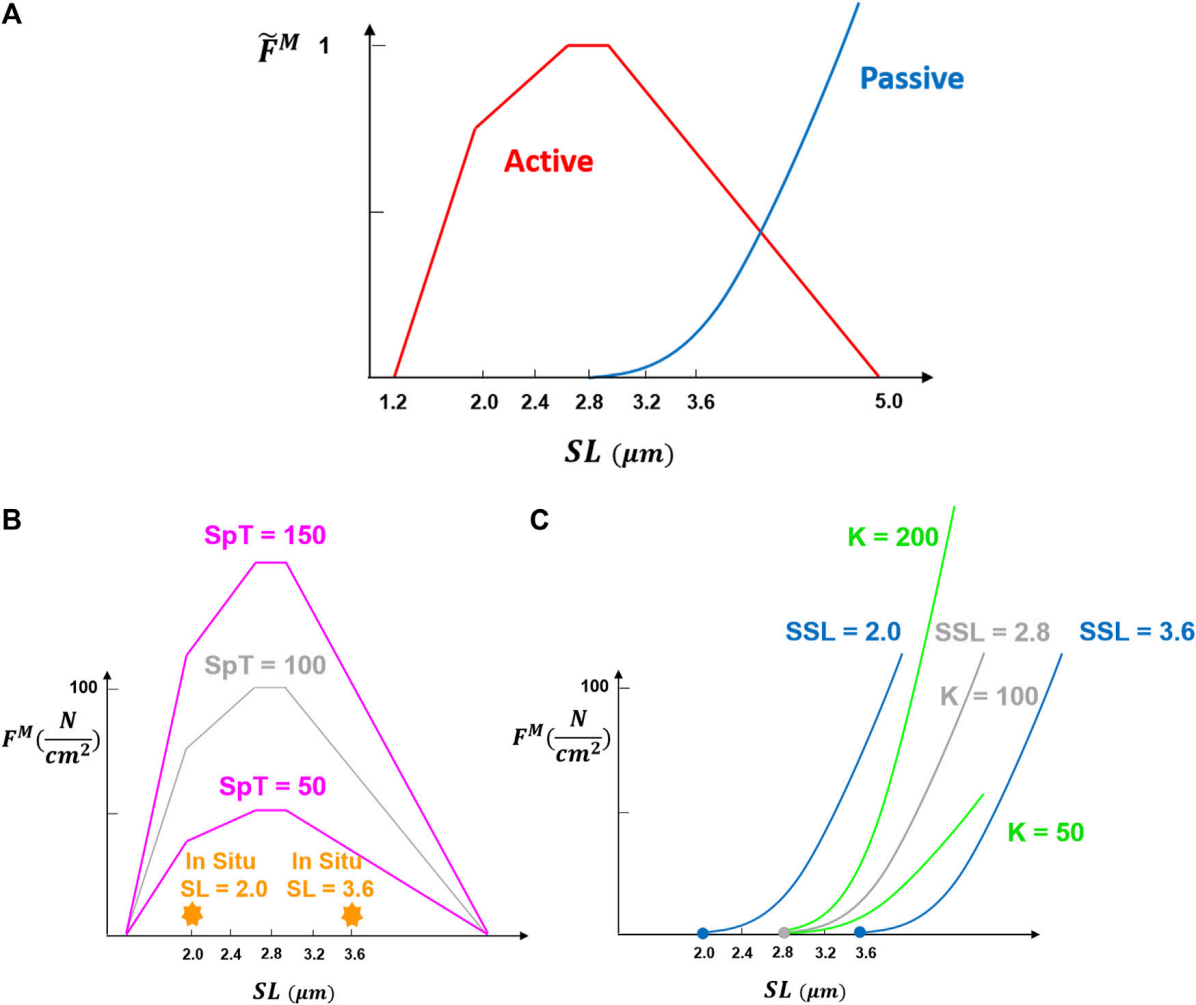
Fundamental muscle force-length curve was adopted from Millard et al. (2013). Normalized muscle force ${\tilde{F}}^{M}$ is equal to 1 at optimum sarcomere length which is assumed to be 2.8 μm in humans. Multiplying ${\tilde{F}}^{M}$ by the specific tension gives the maximum force per unit area a muscle can produce when fully activated which depends on the sarcomere length [graph **(A)**]. The nonnormalized force-length curves may not be the same for all muscles or individuals, and the generated forces could vary with regard to certain parameters including [graph **(B)**] posture-specific *in situ* sarcomere length (SL) and the specific tension (SpT) or [graph **(C)**] slack sarcomere length (SSL) and stiffness scaling factor (K). Note that five different values were considered and tested for each parameter in this study but only two or three representative values for each parameter are shown in this figure. The gray color refers to the values used for the baseline model.

Spinal loading depends upon the biomechanical properties of the paraspinal muscles. These properties include physiological cross-sectional area (PCSA), passive stiffness, slack sarcomere length (SSL, beyond which passive force starts to develop), *in situ* sarcomere length (i.e., sarcomere length measured inside the body for a certain posture), pennation angle, and specific tension (defined as the maximum force per unit area produced by contractile elements) (Figure 1). For example, muscle PCSA has been shown to affect both spinal loading magnitudes (Jamshidnejad and Arjmand, 2015; Malakoutian et al., 2016a) and muscle activation patterns (Bresnahan et al., 2010). The importance of many other muscle parameters to the spine remains unknown, even though they have great importance to the biomechanical functioning of the individual muscles.

The well-known force-length relation of muscle (Figure 1) is included appropriately in only a relatively few optimization-based biomechanical models of the spine [e.g., (Christophy et al., 2012; Bruno et al., 2015; Malakoutian et al., 2016b; Senteler et al., 2016)]. Even in these models, most of the required muscle parameters are either assumed or taken from cadaveric studies. For example, the passive elastic modulus, slack sarcomere length, and specific tension are assumed to be the same for all muscles in these models, while those have been shown to differ between muscle groups or between pathologies (Ward et al., 2009c; Lieber et al., 2003; Smith et al., 2011; Luden et al., 2008; Noonan et al., 2021). Due to the ethical and technical challenges, only a few and limited *in vivo* measurements of these parameters have been made to date (Ward et al., 2009c; Malakoutian, 2021). This might be because the significance of these parameters in spine modeling is not yet fully understood. In fact, there are no studies that have assessed the effect of these different muscle properties on spinal loading within these different models (Christophy et al., 2012; Bruno et al., 2015; Malakoutian et al., 2016b; Senteler et al., 2016).

Therefore, the objective of this study was to explore changes in spinal loading due to variation in the paraspinal muscle parameters, specifically slack sarcomere length, passive stiffness, *in situ* sarcomere length, specific tension, and pennation angle.

## 2 Materials and Methods

We used a recent detailed model of the lumbar spine developed by our group (Malakoutian et al., 2016b), which was based on the model introduced by Christophy et al. (2012) and used ArtiSynth (www.artisynth.org) for physical simulation (Lloyd et al., 2012). In this study, the solution method was enhanced to extend the validation to several activities and postures as described in the following sections.

### 2.1 Geometric Model

The geometry and mechanical properties of the spine model were the same as reported previously (Malakoutian et al., 2016b). The model consisted of five mobile lumbar vertebrae, L1–L5, with the entire thorax rigidly fixed to L1. The sacrum and pelvis were fixed to the ground and the segmental weights of the upper body along with the weights of the head, neck, and arms were all incorporated. The adjacent lumbar vertebrae were connected through massless 6-DOF springs (i.e., six degrees of freedom including three translation and three rotation) with a 6 × 6 stiffness matrix for each lumbar functional spinal unit (FSU, defined as a pair of adjacent vertebrae with connecting ligaments, facet joints, and intervertebral disc). Relative displacements and rotations between two adjacent vertebrae generate restoring forces and moments by the 6-DOF springs which are applied equally but in opposite directions at the centers of the two vertebrae. Due to the paucity of the data in the literature, only diagonal terms of the stiffness matrix were included: translational terms were taken from one study (Gardner-Morse and Stokes, 2004) while the nonlinear formulations for the rotational terms were adopted from another (Heuer et al., 2007b). The effect of Intra-abdominal pressure was modeled as a force applied on the thorax inserted at the center of the diaphragm remaining normal to the diaphragm surface at all postures (Han et al., 2012).

Muscles in the model comprised 210 fascicles, each modeled as a Hill-type musculotendon actuator. PCSA, supine/prone *in situ* sarcomere length, pennation angle, and fiber to tendon ratios were defined for all muscles, as was done by Christophy et al. (2012). These anatomic and biomechanical properties are all involved in muscle force computation as:

$$F_{muscle}=PCSA\times \left(activation\times SpT\times {\tilde{f}}_{active}\left(SL\right)+K\times {\tilde{f}}_{passive}\left(SL\right)\right)\times \cos\alpha \;$$

where K is a constant scaling of the normalized passive curve similar to how specific tension (SpT) acts to normalize the active curve; SL is the sarcomere length calculated from model fiber length and other anatomic properties (as described in Supplementary Material S1 in detail); ${\tilde{f}}_{active}$ and ${\tilde{f}}_{passive}$ are force multipliers as functions of SL obtained from the force-length curves (Figure 1), α is pennation angle and activation of muscle is a decimal varying between 0 and 1.

The normalized force-length and force-velocity curves were taken from the study by Millard et al. (2013) and tendons were assumed to be rigid. The baseline value K was chosen equal to the specific tension in our model.

### 2.2 Solution Method

**FIGURE 2:**
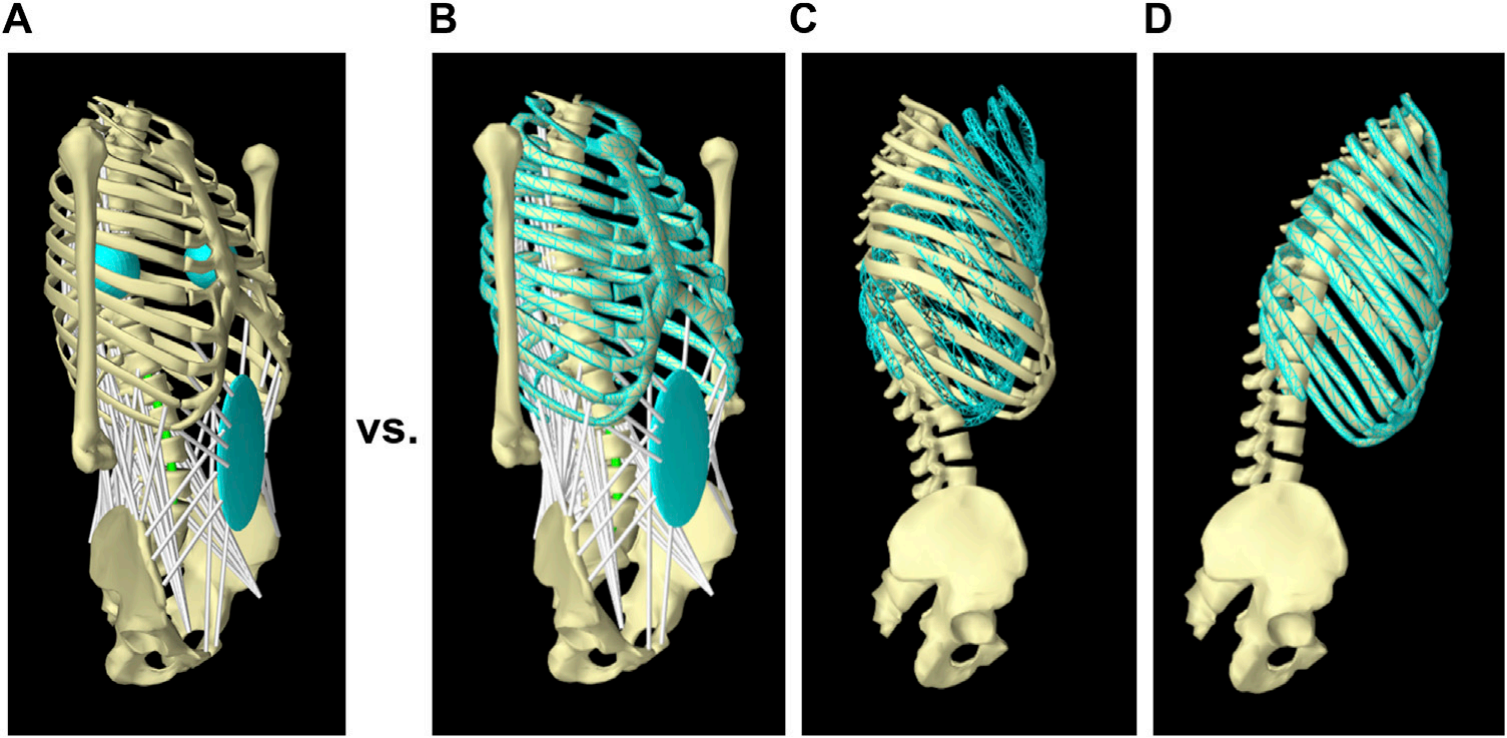
Tracking target frames instead of target points in the new solution method. In the previous model (Malakoutian et al., 2016b), the full trajectory of two symmetric target points located at the right and left of the thorax was assigned as input and tracked by the model **(A)**; while in the new solution method, only rotation of the thorax was given as input **(B and C)** and followed by the model **(D)**.

Our spine model in ArtiSynth used forward dynamics assisted data tracking and optimization to solve the muscle redundancy problem (Malakoutian et al., 2016b; Sagl et al., 2019; Erdemir et al., 2007). The optimization predicts muscle activations to achieve an input trajectory for one or more target points (Figure 2A). In the current model, we set the thorax rotation as an input into the optimization cost function of the model, while the other mobile rigid bodies (i.e., L2–L5) were all able to move freely (Figures 2B–D). This is a better approach than our previous strategy of prescribing the position of a specific set of target points, as it eliminates the sensitivity of the spinal forces to the translational component of the prescribed trajectory observed in other models (Malakoutian et al., 2016b; Eskandari et al., 2019; Dehghan-Hamani et al., 2019; Byrne et al., 2020). The cost function for the optimization was a weighted summation of four terms: the kinematics tracking error, the sum of muscle activations squared, the sum of the square of the difference between the activations of two consecutive time steps, and the sum of FSUs (6-DOF springs) forces squared. The fourth term was added to the optimization cost function to minimize the intervertebral loads (Schultz et al., 1982; Stokes and Gardner-Morse, 2001; Arjmand and Shirazi-Adl, 2006b), and was different from our previous model. Quadratic programming was used to solve for the set of muscle activations that would minimize the total cost function. The weighting terms were determined through the calibration process.

### 2.3 Calibration

The purpose of model calibration is to choose the four weighting terms used for summing the optimization cost functions and to determine the muscle-specific tension. For the computation of muscle forces, the normalized force-length curve and force-velocity curve are scaled by the specific tension, which is defined as the maximum contractile force per unit area a muscle can produce at its optimum length; and is typically assumed to be the same for all muscles in a musculoskeletal model. The specific tension along with the weighting terms for the first three cost functions was determined in our previous model through the simulation of two postures (10° extension and 10° flexion) of an *in vivo* study (Daggfeldt and Thorstensson, 2003). Adopting the same values, we followed a similar calibration approach including two additional postures (−20° extension, 30° flexion) and determined the weighting term for the new FSU load term in the cost function (see Supplementary Material S2 for the details of our calibration approach). Among the three values of 80, 90, and 100 $N/cm^{2}$ , a specific tension of 100 $\;N/cm^{2}$ was again found to produce model results (including maximum producible extension moments) that were closest to experimentally reported values (Daggfeldt and Thorstensson, 2003). Any simulation with a tracking error of more than 1° from the prescribed thorax position was not considered converged and was rejected in this study (identified as red stars in figures).

### 2.4 Validation

For validation of the new solution method, we compared the prediction of our model for L4-L5 IDP to the L4-L5 IDP measured *in vivo* for five symmetric activities (Wilke et al., 2001): 1) 19° extension, 2) upright standing, 3) and 4) holding a crate of 190 N close and far from the chest (25 and 55 cm anterior to the L5-S1 disc), and 5) 36° flexion. The output of our model was the forces and moments on each FSU (6-DOF spring). We performed a post-analysis to compute the IDP values as the sum of IDP resulting from the compressive force and the IDP from the flexion/extension moment. We assumed that shear force did not influence IDP (Frei et al., 2001). The IDP associated with the compressive force, $IDP_{F_{axial}}$ , was calculated as:

$$IDP_{F_{axial}}=\frac{F_{axial}\times 0.85}{Disc\;Area\times 0.66}$$

where 0.85 is considered as the intervertebral disc share of the compressive force on the FSU, $F_{axial}$ (Nachemson, 1960; Ghezelbash et al., 2020), 0.66 is the correction factor for the nucleus area (Nachemson, 1960; Dreischarf et al., 2013), and the intervertebral disc area was set to 18 $cm^{2}$ (Wilke et al., 2001). Notably, the mass (70 kg) and height (174 cm) of the volunteer in the study by Wilke et al. matched well with those set for our model (i.e., 71 kg and 170 cm). For calculation of the IDP associated with the flexion moment, $IDP_{M_{flexion}}$ , and extension moment, $IDP_{M_{extension}}$ , a linear fit to the data of an *in vitro* study (Heuer et al., 2007a) led to the following relations:

$$IDP_{M_{flexion}}=M_{flexion}\times 0.036\frac{MPa}{N.m},$$

$$IDP_{M_{extension}}=M_{extension}\times 0.018\frac{MPa}{N.m},$$

where $M_{flexion}$ and $M_{extension}$ were the magnitudes of the flexion and extension moments applied to the FSU.

The predicted paraspinal muscle activation for the upright standing and 36° flexion postures were additionally contrasted against the *in vivo* electromyographic (EMG) findings in the literature (Peach et al., 1998; McGill et al., 1999).

Two postures of 10° extension and 40° flexion were also simulated to compare the predicted rotations of the vertebrae against those measured for 50 healthy men (Wong et al., 2004).

### 2.5 Study Design for Investigating Impact of Muscle Parameters

The model used for validation (Section 2.4) was considered as the baseline model and all values for muscle parameters in that model were considered baseline values. Using that model, we examined the changes in L4-L5 IDP in response to variation in each of the following five muscle parameters (one parameter at a time): slack sarcomere length, passive stiffness, supine/prone *in situ* sarcomere length, specific tension, and pennation angle. For two of the parameters, specifically passive stiffness and supine/prone *in situ* sarcomere length, an extra set of simulations was performed when the slack sarcomere length was 0.4 μm shorter than its baseline value (to explore possible interplays between these parameters). All simulations were performed for two postures of upright standing and 36° flexion.

Baseline values for three of the muscle parameters were the same in all muscles: SSL 2.8 μm, scaling factor for the normalized passive stiffness curve (K) 100 $N/cm^{2}$ , and specific tension (SpT) 100 $N/cm^{2}$ . Baseline values for the other two muscle parameters, supine/prone *in situ* sarcomere length (InSituSL) and pennation angle (PenAng), differed amongst muscle groups and were taken from Table 3-3 in Malakoutian, 2014.

The baseline value for the slack sarcomere length in the curves used (Millard et al., 2013) was the optimum sarcomere length, which is assumed to be 2.8 μm in humans (Delp et al., 2001). Although a slack sarcomere length value at the whole muscle level is desired, that has never been measured for paraspinal muscles. The slack sarcomere length values measured for human paraspinal muscles range between 1.8 and 2.8 μm microns for individual muscle fibers and fiber bundles (Malakoutian, 2021) and thus for our study, we tested five different values of 2.0, 2.4, 2.8 (baseline), 3.2, and 3.6 μm (Figure 1C). Although larger values of 3.2 and 3.6 μm have not been reported yet, those were included in case such values are observed in future studies for a certain pathology or when measurements are made at the whole muscle level.

The normalized passive curve was scaled by a constant K of 100 $N/cm^{2}$ equal to the selected specific tension for the baseline model. The quintic Bezier splines (Millard et al., 2013) for the muscle force-length curve-fitting provide a high order of continuity and smoothness, but their formulation is not intuitive. To get a sense of muscle stiffness, the tangent modulus at 10, 30, 50, and 70% strains were 0.26, 1.01, 2.45, and 2.86 MPa, respectively, for the baseline model. To study changes in spinal loading due to variation in muscle stiffness, the normalized passive curve was scaled by five different *K* values of 10, 50, 100 (baseline), 500, and 1000 $N/cm^{2}$ which resulted in passive stiffnesses close to the ranges reported in the literature for fiber bundles (Malakoutian, 2021; Lieber et al., 2003) and whole muscles (Ward et al., 2020).

The supine/prone *in situ* sarcomere length and pennation angle of the muscles in our baseline model were taken from both *in vivo* and cadaveric studies in the literature and differed between muscle groups [see Table 3-3 in Malakoutian, 2014]. *In situ* sarcomere length for human paraspinal samples measured in supine/prone posture exhibits values between 1.9 and 3.6 μm (Malakoutian, 2021; Ward et al., 2009a; Bayoglu et al., 2017); thus, we tested five different values of 2.0, 2.4, 2.8, 3.2, 3.6 μm . For pennation angle, the values ranged between 0° and 14° for the paraspinal muscles. To get a better understanding of its impact on IDP, especially given the measured values of up to 30° for some other muscles of the human body (Ward et al., 2009a), we tested a set of values of 0°, 7.5°, 15°, 22.5°, and 30°. For supine/prone *in situ* sarcomere length and passive stiffness, in particular, we ran all the simulations once with a slack sarcomere length equal to 2.8 μm (baseline) and the other time with setting the slack sarcomere length of the targeted muscles to 2.4 μm, to explore the possible interplay between these parameters.

The specific tension measured experimentally for human single fibers rarely exceeds an average value of ∼40 $N/cm^{2}$ (Cristea et al., 2008). However, the majority of biomechanical models have chosen values of 80–100 $N/cm^{2}$ to be able to perform heavy work activities requiring large muscle forces and to be validated against the *in vivo* data (Schultz et al., 1982; Van Dieën and Kingma, 1999; de Zee et al., 2007; Bresnahan et al., 2010; Bruno et al., 2015; Ignasiak et al., 2016; Bayoglu et al., 2019). We considered specific tension values of 5, 10, 25, 50, 100 (baseline), and 150 $N/cm^{2}$ , to investigate their influence on the L4-L5 IDP. The range of 5–50 N/cm^2^ has been measured biologically at the muscle fiber level (Cristea et al., 2008), but 100 and 150 have been used only in biomechanical models (Burkhart et al., 2018; Bruno et al., 2015; Holzbaur et al., 2005).

The changes in each muscle parameter were applied in four different scenarios: scenario 1, MUL, involved changes only applied to the multifidus; scenario 2, EXT, involved changes applied to extensor muscles including multifidus, longissimus thoracis, iliocostalis lumborum, and quadratus lumborum; scenario 3, EXT + PS, involved changes applied to extensor muscles as well as psoas; and scenario 4, ALL, involved changes made to all 210 muscle fascicles in the model. These scenarios were defined as simulating plausible scenarios related to a certain pathology or surgical intervention. For example, with genetic pathologies, all muscles may be affected and present higher stiffness values; while for changes after spinal surgeries only multifidus or all those directly attached to the spine (i.e., extensors and psoas) may be involved.

## 3 Results

### 3.1 Validation

**FIGURE 3:**
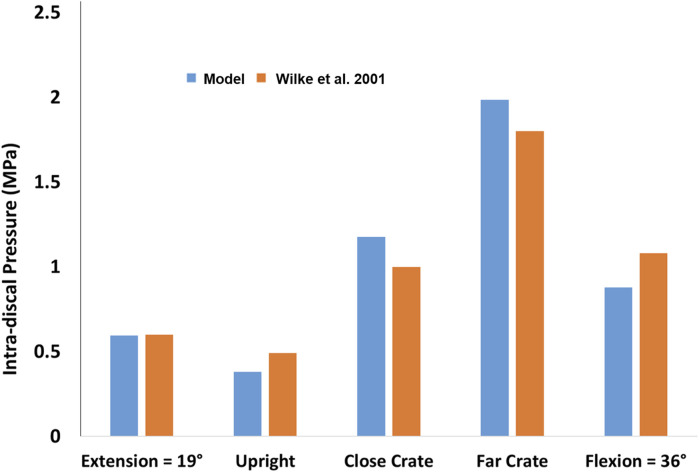
Comparison between the predicted L4-L5 IDP by the model and those measured *in vivo* for five different activities (Wilke et al., 2001).

The resultant compressive and shear forces along with the flexion moments for all lumbar vertebral levels are presented for the five symmetric activities performed by the baseline model (Table 1). The corresponding IDP with forces and moments at the level L4-L5 compared well with the *in vivo* IDPs (Figure 3), such that a linear fit to the predicted L4-L5 IDPs by the model and those measured *in vivo* resulted in a coefficient of determination of 0.98.

**TABLE 1:** Predicted compressive forces, shear forces, and sagittal plane moments at all vertebral levels by the model for the five different activities performed by the subject of the *in vivo* study (Wilke et al., 2001).

Spinal level	Activity	Compressive force (N)	Anterior shear force (N)	Sagittal plane moment (Nm)
L1-L2	1–19° extension	355	−351	−6.35
	2–Upright standing	467	−105	−1.17
	3–Holding crate close	1456	−163	−1.11
	4–Holding crate far	2652	−240	−8.53
	5–36° flexion	396	462	13.59
L2-L3	1–19° extension	456	−289	−6.01
	2–Upright standing	514	−60	−0.99
	3–Holding crate close	1841	63	−5.06
	4–Holding crate far	2976	−130	−7.96
	5–36° flexion	568	430	11.53
L3-L4	1–19° extension	549	−183	−6.12
	2–Upright standing	533	−2	−0.56
	3–Holding crate close	1802	350	−2.8
	4–Holding crate far	2998	318	−5.25
	5–36° flexion	729	341	9.56
L4-L5	1–19° extension	670	57	−6.98
	2–Upright standing	529	107	0.26
	3–Holding crate close	1646	653	0.65
	4–Holding crate far	2798	763	0.71
	5–36° flexion	797	324	8.88
L5-S1	1–19° extension	671	524	−7.38
	2–Upright standing	469	235	1.73
	3–Holding crate close	1432	803	6.15
	4–Holding crate far	2571	1042	6.57
	5–36° flexion	813	403	8.41

The crate weighed 190 N. An anterior shear force with a negative value was directed posteriorly. A positive value for the sagittal plane moment causes the upper vertebra of the FSU to flex.

The activity of the extensor and abdominal muscles also compared well with the EMG findings in the literature. In 36° flexion, the activation level of extensor muscles dropped such that the sum of active forces in extensor muscles was 44 N in our model compared to 206 N in upright standing, mimicking the well-known flexion-relaxation phenomenon (Colloca and Hinrichs, 2005; Peach et al., 1998; McGill et al., 1999). The occurrence of this phenomenon is attributed to the development of forces in passive tissues including the extensor muscles (from 0 N in standing to 334 N in 36° flexion in our model), as suggested in previous studies (Colloca and Hinrichs, 2005). On the other hand, the active forces in abdominal muscles increased from 4 N in upright standing to 371 N in 36° flexion, which is also in harmony with the recorded increase in EMG of human abdominal muscles in flexion (Peach et al., 1998; McGill et al., 1999).

**FIGURE 4:**
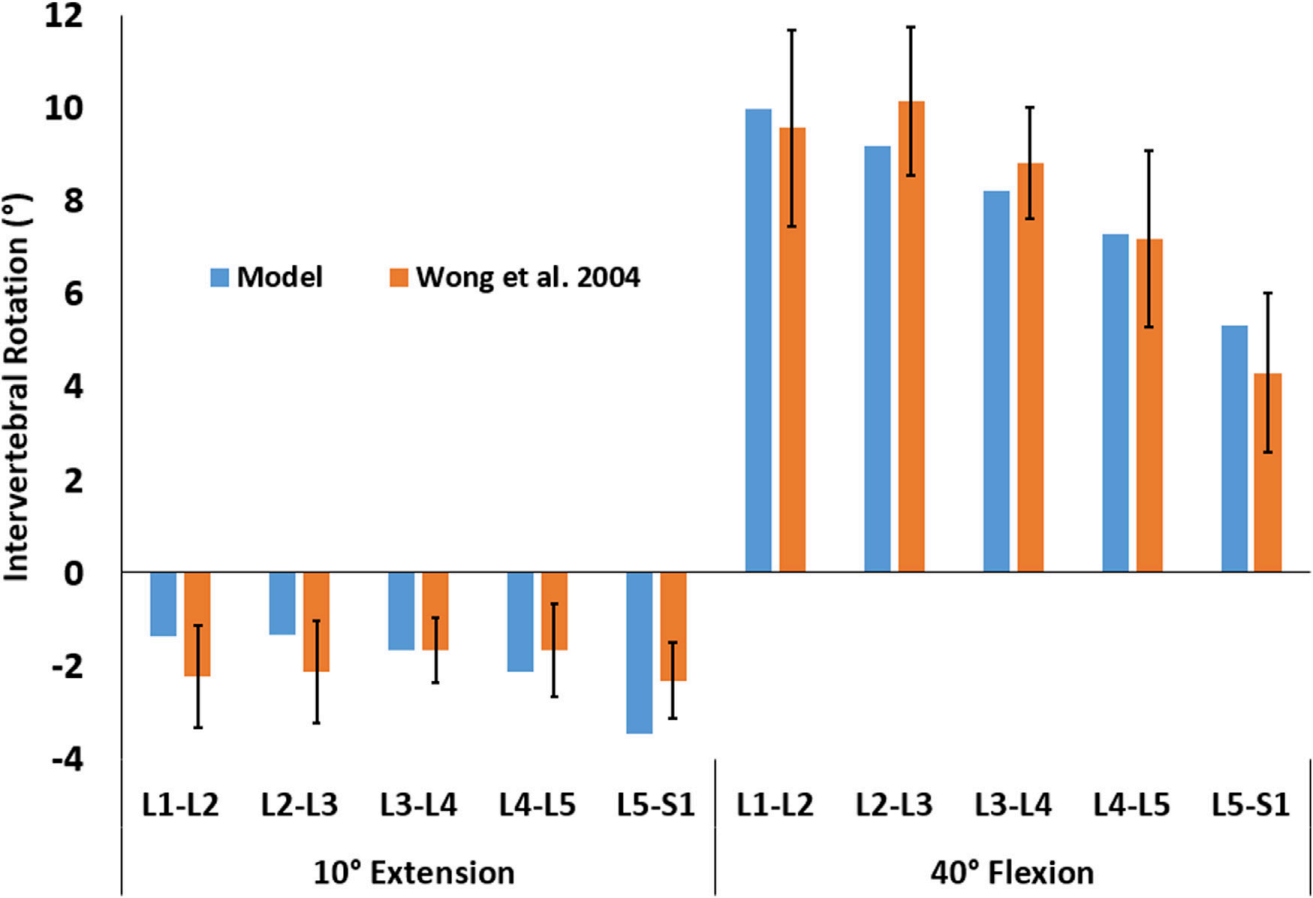
Intervertebral rotations for two activities of 10° extension and 40° flexion were predicted by the model (blue) and observed in 50 male subjects (orange, Wong et al., 2004). The error bars represent the mean ± one standard deviation.

The rotation of the thorax was dictated by the user, but the trajectories of the other vertebrae were free (i.e., were not determined with a predefined function). The intervertebral rotations from upright standing for both 10° extension and 40° flexion fell within the range observed for 50 healthy men (Wong et al., 2004; Figure 4).

### 3.2 Impact of Muscle Parameters on L4-L5 Intradiscal Pressure

All muscle parameters except the pennation angle had a dramatic impact on the predicted L4-L5 IDP in both standing and flexion activities.

#### 3.2.1 Slack Sarcomere Length

**FIGURE 5:**
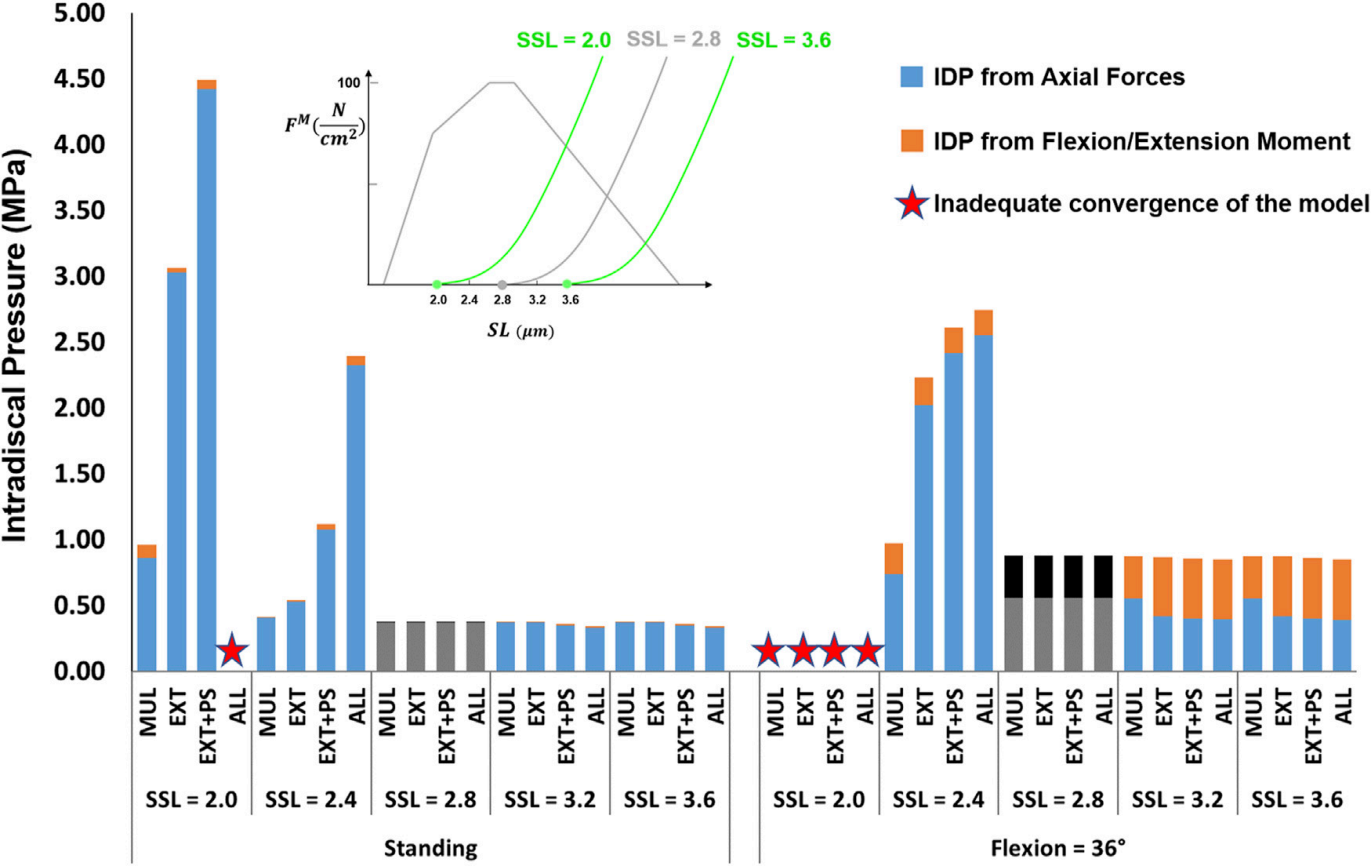
The effect of different slack sarcomere length (SSL) values on L4-L5 IDP in upright standing and 36° flexion for four scenarios: changes applied to multifidus (MUL), extensor muscles (EXT), extensor muscles, and psoas (EXT + PS), and all 210 muscles in the model (ALL). Grey/black bars represent the baseline values.

The influence of slack sarcomere length on the IDP was greatest when it was set to 2.0 μm or 2.4 μm (Figure 5, note that slack sarcomere length of 2.8 μm is the baseline value). For example, changing the slack sarcomere length of the multifidus to 2.0 μm and keeping the slack sarcomere length equal to 2.8 μm for the other muscles, doubled the IDP in standing. This occurrence was due to the development of passive forces in the multifidus, whose sarcomere length was 2.27 μm in standing. The IDP was seven times and 10 times larger in standing when the slack sarcomere length of EXT and EXT + PS were also set to 2.0 μm, respectively. The model was not able to reach 36° flexion for any of these cases due to substantial passive forces that would have been developed in that posture. The same but milder trend was observed when slack sarcomere length was set to 2.4 μm in standing; while in flexion, the IDP even tripled (increased from a baseline value of 0.88–2.6 MPa). For slack sarcomere length values larger than 2.8 μm, the total IDP did not change, although the distribution between the IDP from the compressive force and the IDP from flexion/extension moment changed somewhat.

#### 3.2.2 Passive Stiffness

**FIGURE 6:**
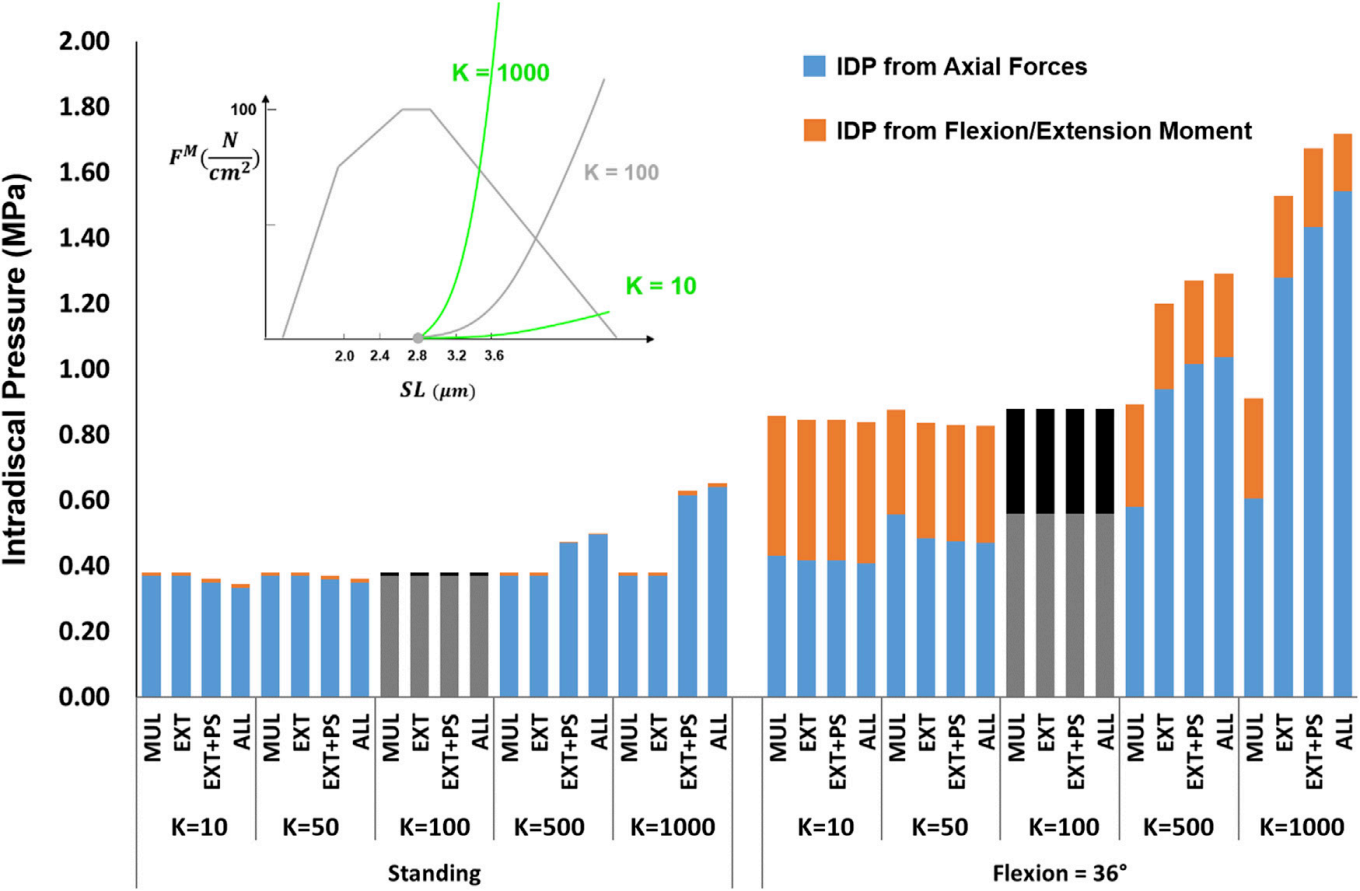
The effect of different passive force-length curve scaling constants (K in $\frac{N}{cm^{2}}$ ) on L4-L5 IDP in upright standing and 36° flexion for four scenarios: changes applied to multifidus (MUL), extensor muscles (EXT), extensor muscles, and psoas (EXT + PS), and all 210 muscles in the model (ALL). Grey/black bars represent the baseline values.

**FIGURE 7:**
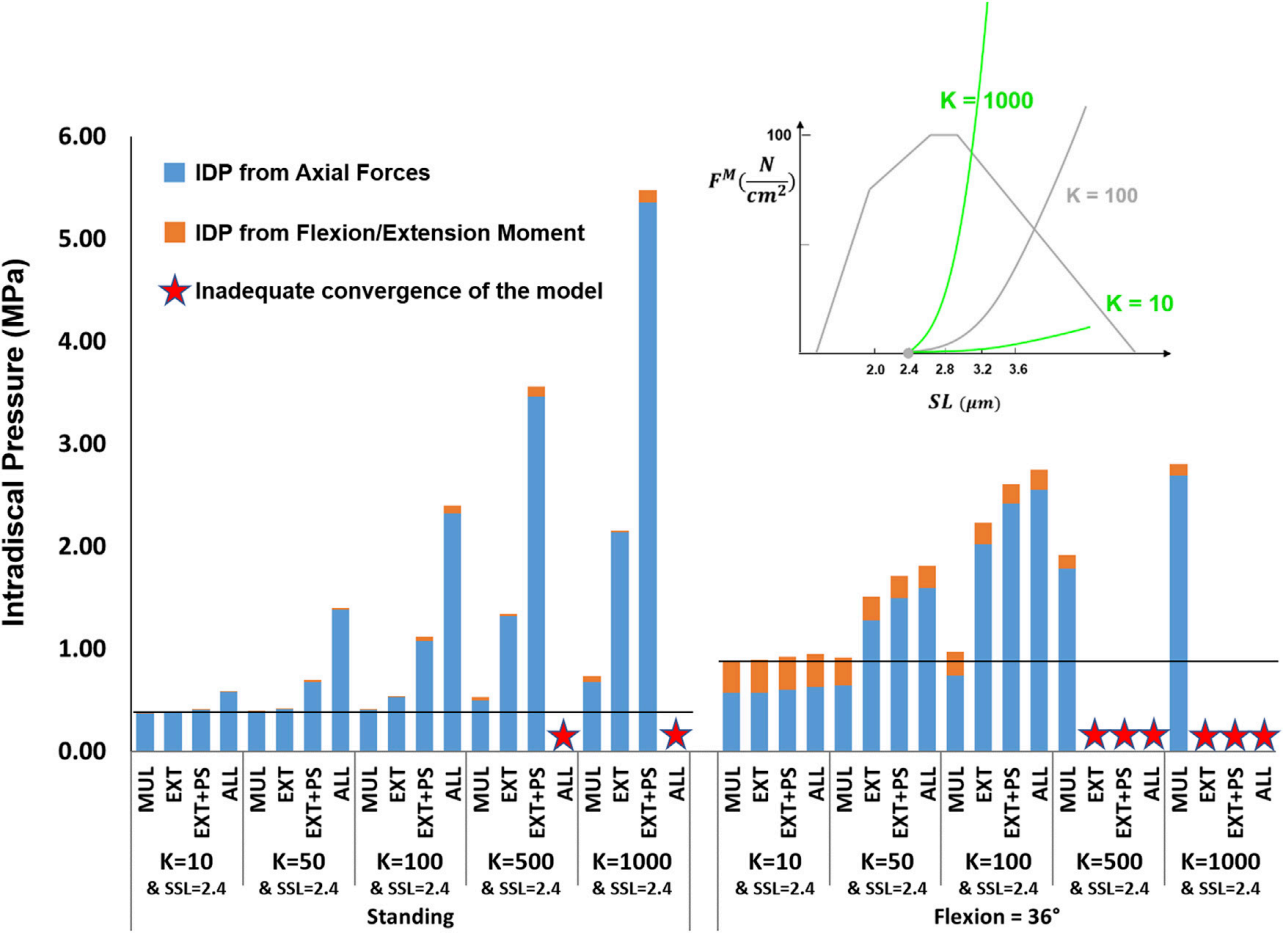
The effect of different passive force-length curve scaling constants (K in $\frac{N}{cm^{2}}$ ) combined with a slack sarcomere length (SSL) of 2.4 μm to the targeted muscles on L4-L5 IDP in upright standing and 36° flexion for four scenarios: changes applied to multifidus (MUL), extensor muscles (EXT), extensor muscles and psoas (EXT + PS), and all 210 muscles in the model (ALL). The black horizontal lines represent the baseline values.

The effects of passive stiffness and supine/prone *in situ* sarcomere length on IDP were both dependent on the slack sarcomere length. When slack sarcomere length was 2.8 μm, reducing the passive stiffness to half or even one-tenth of the baseline value did not change the IDP by more than 20% (Figure 6). However, when passive stiffnesses were increased to five times or 10 times greater than the baseline, the IDP increased considerably for most scenarios, especially in flexion (Figure 6). When slack sarcomere length was set to 2.4 μm for the targeted muscles whose stiffness also changed, for all stiffness scaling factors the IDP changed dramatically both in standing and flexion and its extent depending on what muscles were manipulated (Figure 7). For example, a five or 10 fold increase in multifidus stiffness, combined with setting its slack sarcomere length to 2.4 μm, elevated the IDP in flexion from ∼1 to ∼2 or ∼3 MPa. This IDP increase was a result of multifidus passive forces developed after its sarcomeres lengthened from 2.27 μm (on average) in standing, passed the slack sarcomere length (i.e., 2.4 μm) in 11° flexion and reached 3.01 μm (leading to ∼25% strain in multifidus) in 36° flexion.

#### 3.2.3 *In Situ* Sarcomere Length

**FIGURE 8:**
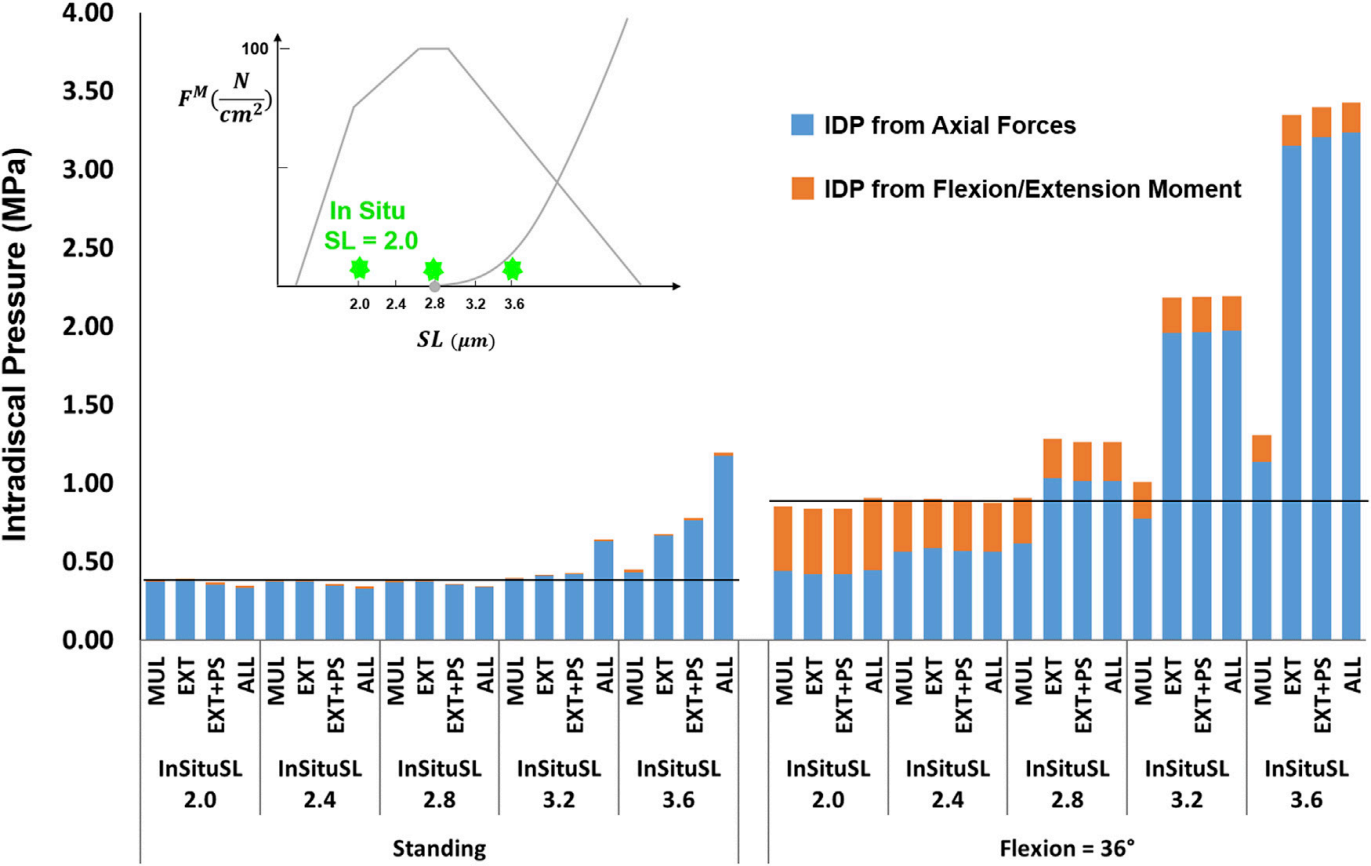
The effect of different supine/prone *in situ* sarcomere length on L4-L5 IDP in upright standing and 36° flexion for four scenarios: changes applied to multifidus (MUL), extensor muscles (EXT), extensor muscles, and psoas (EXT + PS), and all 210 muscles in the model (ALL). The black horizontal lines represent the baseline values.

**FIGURE 9:**
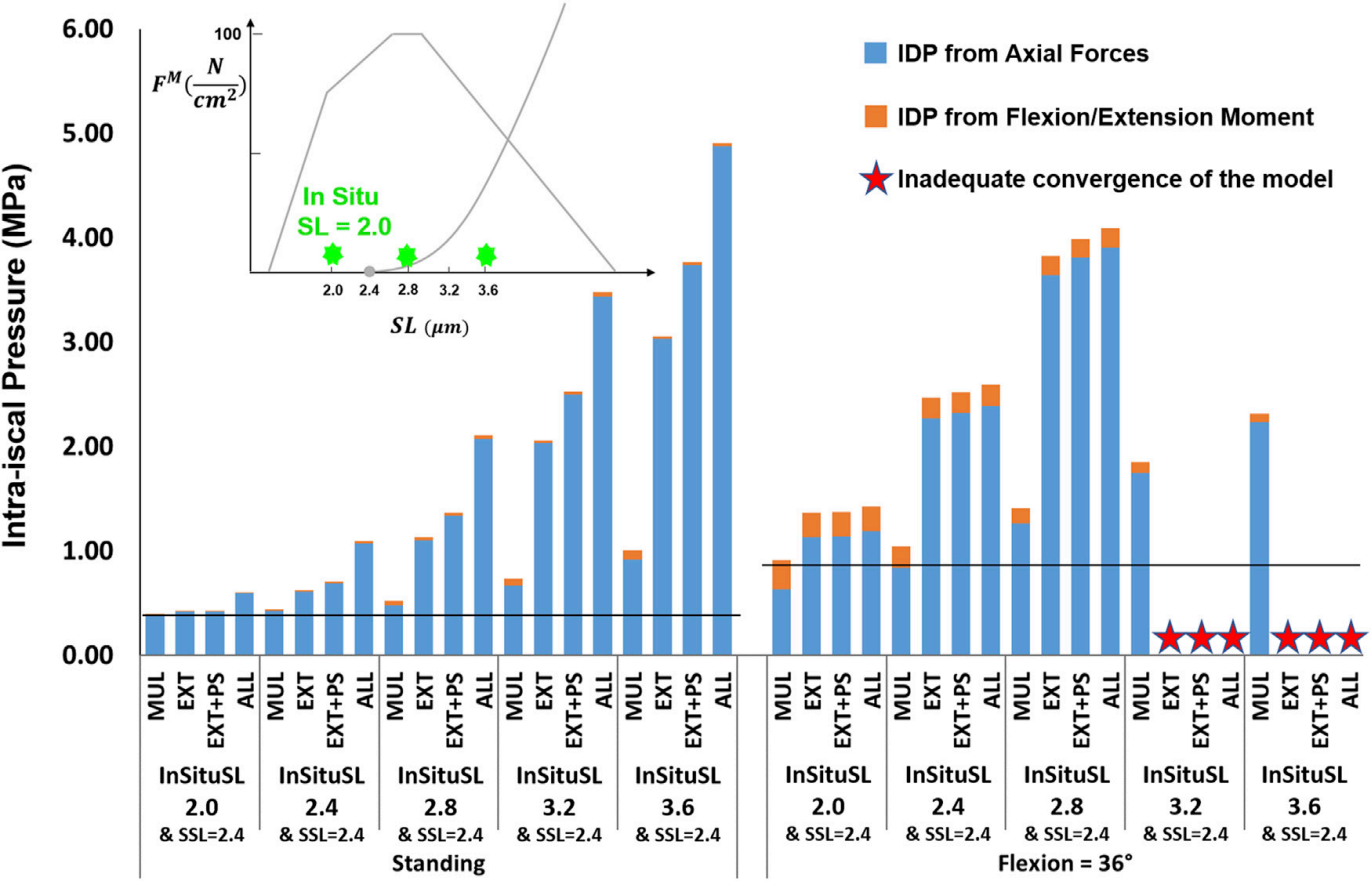
The effect of different supine/prone *in situ* sarcomere lengths combined with a slack sarcomere length (SSL) of 2.4 μm to the targeted muscles on L4-L5 IDP in upright standing and 36° flexion for four scenarios: changes applied to multifidus (MUL), extensor muscles (EXT), extensor muscles and psoas (EXT + PS), and all 210 muscles in the model (ALL). The black horizontal lines represent the baseline values.

For the supine/prone *in situ* sarcomere length values of 2.0 and 2.4 μm when slack sarcomere length was 2.8 μm, only small differences in IDP were observed (Figure 8). For greater supine/prone *in situ* sarcomere lengths, however, the increase in the IDP was large, especially in flexion, where sarcomere lengths exceeded the slack sarcomere length. For example, when the supine/prone *in situ* sarcomere length of the entire group of extensor muscles was 3.6 μm, the IDP increased by 79% (reaching 0.68 MPa from 0.38 MPa) in standing and by 380% (reaching 3.34 MPa from 0.88 MPa) in flexion. When slack sarcomere length was set to 2.4 μm for the targeted muscles whose supine/prone *in situ* sarcomere length was also changed, much larger increases in IDP occurred for all supine/prone *in situ* sarcomere length values (Figure 9). For example, when multifidus was manipulated alone to a slack sarcomere length of 2.4 μm and a supine/prone *in situ* sarcomere length of 3.6 μm compared to when it is set to 2.0 μm, the IDP doubles in both standing and flexion.

#### 3.2.4 Specific Tension

**FIGURE 10:**
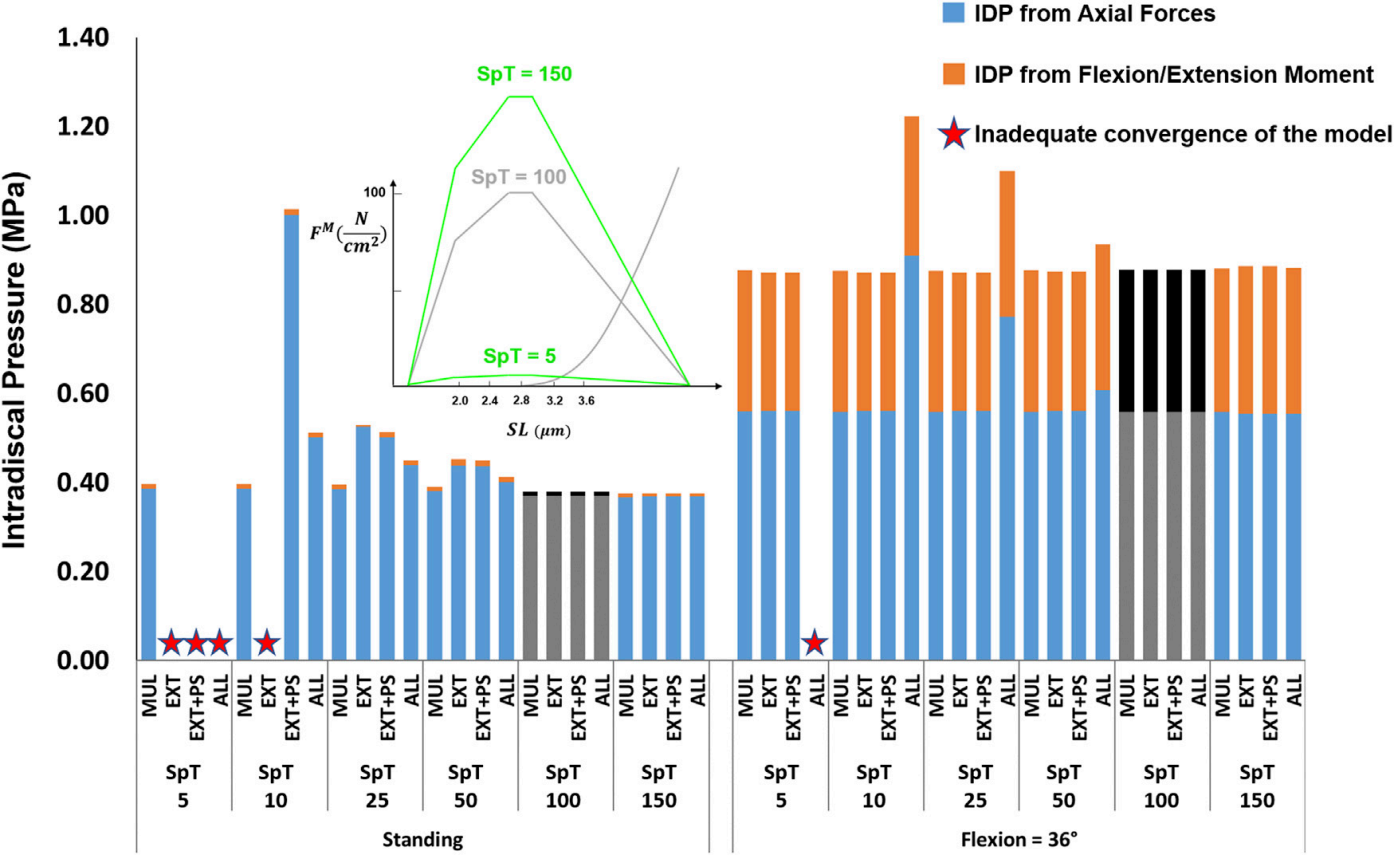
The effect of different specific tension (SpT) values on L4-L5 IDP in upright standing and 36° flexion for four scenarios: changes applied to multifidus (MUL), extensor muscles (EXT), extensor muscles and psoas (EXT + PS), and all 210 muscles in the model (ALL). Grey bars represent the baseline values.

The studied values for the specific tension had a minimal influence on the IDP in flexion, except for the scenario where the changes were applied to all muscles, which increased the IDP by 39% when the specific tension was set to one-tenth of its baseline value (Figure 10). For upright standing, the increase in specific tension also had little effect on the IDP. However, when the specific tension was reduced to half or a quarter of its baseline value, the IDP manifested an increasing trend, with the largest increases occurring when the changes were made to the extensor muscles only. The model was not able to achieve the upright posture when the specific tension was decreased for the extensor muscles to 10% of the baseline value, or 5% of the baseline value for all scenarios, except for when the change was made to multifidus only.

#### 3.2.5 Pennation Angle

**FIGURE 11:**
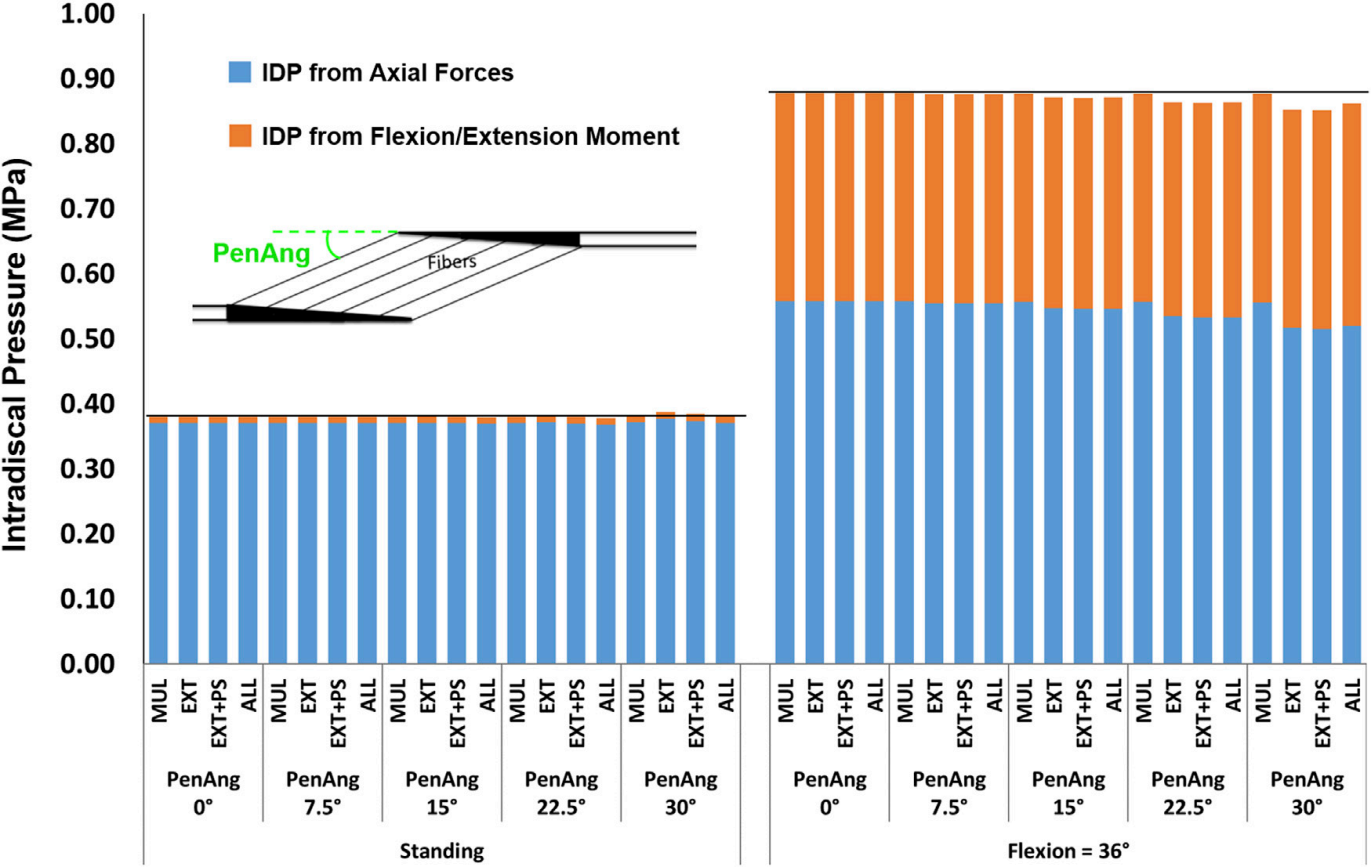
The effect of different pennation angles on L4-L5 IDP in upright standing and 36° flexion for four scenarios: changes applied to multifidus (MUL), extensor muscles (EXT), extensor muscles, and psoas (EXT + PS), and all 210 muscles in the model (ALL). The black horizontal lines represent the baseline values.

The influence of pennation angle on the overall IDP for both standing and flexion was negligible (Figure 11).

## 4 Discussion

Musculoskeletal models serve as promising tools for gaining knowledge of spinal loading during various daily activities. The accuracy of their predictions, however, relies on the input variables, including the biomechanical properties of the muscle. The significance of the muscle force-length curve and the parameters associated with it is not yet clearly known for spinal loading, which is probably why only a relatively few optimization-based models have included that appropriately in their solution methods [e.g., (Christophy et al., 2012; Bruno et al., 2015; Malakoutian et al., 2016b; Senteler et al., 2016)]. The predicted muscle activity, intervertebral rotations, and the L4-L5 IDP in our improved model were in excellent agreement with the corresponding *in vivo* measurements (Wilke et al., 2001; Wong et al., 2004; Peach et al., 1998; McGill et al., 1999). The validated model was used to investigate the variation of the L4-L5 IDP to a range of muscle parameters. The predictions revealed the strong influence of the slack sarcomere length, passive stiffness, *in situ* sarcomere length, and specific tension on spinal loading. The reasons for these observations and their implications will be explored in the following paragraphs in the discussion.

The analysis performed in this study revealed the importance of the muscle force-length curve, including both passive and active components, on spinal loading. We observed a substantial influence of slack sarcomere length, passive stiffness, supine/prone *in situ* sarcomere length, and specific tension on spinal loading, often with interesting interplays between the parameters. For example, in upright standing the values less than 100 $\frac{N}{cm^{2}}$ for K (i.e., the passive-curve constant) did not make any difference to the prediction of the L4-L5 IDP for the baseline slack sarcomere length (i.e., 2.8 μm). However, for the shorter slack sarcomere length (i.e., 2.4 μm), an increase in muscle passive stiffness from *K* = 10 to 50 and 100 $\frac{N}{cm^{2}}$ , increased the IDPs due to passive forces being generated in those muscles and thereby increasing joint forces (Figures 6, 7). A similar intertwined relationship was evident for slack sarcomere length and supine/prone *in situ* sarcomere length (Figures 8, 9) and is expected for passive stiffness and supine/prone *in situ* sarcomere length. This is because for shorter supine/prone *in situ* sarcomere lengths the passive curve does not get involved, therefore changing the stiffness does not make any difference; while for larger supine/prone *in situ* sarcomere lengths, passive forces have already developed thus their values depend on the passive stiffness. Despite the critical importance of these parameters, not enough is known about them in the literature, especially on how they change/adapt together under different conditions (e.g., in different spine pathologies).

The simulated values for all muscle parameters studied herein–slack sarcomere length, supine/prone *in situ* sarcomere length, passive stiffness, specific tension, and pennation angle were relevant based upon the limited human muscle measurements and the biomechanical models in the literature. For example, the supine/prone *in situ* sarcomere lengths for most paraspinal muscles in spine models are taken from cadaveric studies (Ward et al., 2009a; Delp et al., 2001; Bayoglu et al., 2017). While the large variation between these measurements in individuals (Table 2) may be an artifact of the postures in which the cadavers were embalmed, it may reflect natural differences between humans or their pathologies. Prone *in situ* sarcomere length of multifidus and longissimus have been measured *in vivo* with observed ranges for individual measurements between 1.9 and 3.4 μm (Ward et al., 2009b; Malakoutian, 2021). Those measurements were obtained through specialized biopsy clamps and were taken from patients undergoing spinal surgery. Less invasive measurement of this parameter in healthy individuals has become feasible recently (Sanchez et al., 2015) but has not yet been used for paraspinal muscles.

**TABLE 2:** Difference in lumbar spine models with regard to the sources of supine/prone *in situ* sarcomere length values taken from the literature.

Models	Supine/prone *in situ* sarcomere length (μm)							
	MF	IL	LT	QL	Ps	EO	IO	RA
Christophy et al. (2012), Bruno et al. (2015), Malakoutian et al. (2016b), Our baseline model	2.27	2.37	2.31	2.38	3.11	2.83	2.83	2.83
	[W-MF]	[D]	[D]	[D]	[W]	[D]	[D]	[D]
Bayoglu et al. (2017)	3.35	2.81	3.57	2.84	2.6	2.82	2.14	2.72
	[B]	[B]	[B]	[B]	[B]	[B]	[B]	[B]

[W-MF], (Ward et al., 2009b); [W], (Ward et al., 2009a); [D], (Delp et al., 2001); [B], (Bayoglu et al., 2017); MF, multifidus; IL, iliocostalis lumborum; LT, longissimus thoracis; QL, quadratus umborum; Ps, psoas; EO, external oblique; IO, internal oblique; RA, rectus abdominis.

Experimental measurement of slack sarcomere length, passive stiffness, and specific tension requires fresh muscle tissue. slack sarcomere length and passive stiffness of human paraspinal muscles were measured through biopsies collected intraoperatively (Ward et al., 2009c; Malakoutian, 2021). Those measurements were performed on muscle single fibers and fiber bundles (∼10–20 fibers ensheathed in their connective tissue), with the slack sarcomere length exhibiting a range between 1.8 to 2.8 μm for the individual data points. The slack sarcomere length of interest for modeling studies should be measured at the fascicle (∼500 fibers) or whole muscle level. Due to technical challenges, measurement of slack sarcomere length or even passive stiffness and specific tension in spine muscles has never been done for humans or any other species at the fascicle or whole muscle level. Given these data, the slack sarcomere length range studied herein of 2.0 μm through 3.6 μm seems relevant when considering the possibility of larger values if measurements were made at the whole muscle level or for spine pathologic patients.

A similar argument can be made for passive stiffness. In a recent study on rabbits (Ward et al., 2020), it was demonstrated for elastic modulus of lower extremity muscles to increase nonlinearly from smaller scales to larger ones. For example, at 30% strain, the elastic modulus of the extensor digitorum was ∼30, ∼40, ∼260, and ∼7500 kPa, at the fiber, fiber bundle, fascicle, and whole muscle levels, respectively. No study to date has measured the whole muscle stiffness of paraspinal muscles. Interestingly, however, it was observed that at the bundle level for humans (Malakoutian, 2021), individual measurements for stiffness varied between 6 kPa to above ∼2,000 kPa.

Measurement of specific tension is less challenging as it may suffice to be measured at the fiber level only (Winters et al., 2011; Noonan et al., 2021), but it has never been done for human paraspinal muscles. The specific tension for human single fibers in muscles tested to date ranges between ∼10 and ∼40 $N/cm^{2}$ (Luden et al., 2008; Cristea et al., 2008), with the higher value measured in vastus lateralis of world-class sprinters (Cristea et al., 2008). Surprisingly, most lumbar spine models required a specific tension of between 80 and 100 to be able to perform the heavy work activities requiring large muscle forces (Schultz et al., 1982; Van Dieën and Kingma, 1999; de Zee et al., 2007; Bresnahan et al., 2010; Bruno et al., 2015; Ignasiak et al., 2016; Bayoglu et al., 2019). Such a range has been shown to be patient-specific as verified experimentally at the macroscopic level (Burkhart et al., 2018). However, why physiological values for specific tension measured at the muscle fiber level (microscopic level) are not sufficient for biomechanical models remains unanswered.

Inclusion of muscle dynamics and force-length properties is more straightforward for optimization-based models using a forward dynamics approach (Erdemir et al., 2007), but it can be done for those using inverse dynamics also, through the addition of further constraints on muscle forces (Happee, 1994). Using the inverse dynamics approach, input kinematics and external forces are used to calculate the moments at each vertebral level. The moment at each level is distributed between the muscles crossing that level commonly through an optimization technique where the sum of muscle forces/stresses to a certain power is minimized. While passive muscle forces are ignored by some models, those including them subtract the passive component of muscle force (generated depending on model position) from the predicted muscle force to obtain the active force component. These active forces should never exceed the maximum force that a given muscle can generate. This maximum active force is dependent on the length of the muscle. For low moment-demand activities (e.g., upright standing), where muscle activations do not approach their maximum, there is little risk of a model predicting a muscle force to exceed its maximum. However, for activities with larger moment demands, not constraining the force values to within their length-dependent maximum could lead to unrealistic predictions (Erdemir et al., 2007). Without knowledge of the *in situ* sarcomere length for a certain posture, the normalized force-length curve cannot be used appropriately to obtain the length-dependent muscle maximum isometric force (see Supplementary Material S1). Those models that incorporated the force-length curve without knowledge of the *in situ* sarcomere length had to make assumptions, usually implying that the *in situ* sarcomere length at a certain neutral posture (supine, prone, or upright standing) for all muscles was the same or equal to the slack length, while recent cadaveric and *in vivo* studies have revealed large variations for supine/prone *in situ* sarcomere length between spine muscles, and also that optimal length does not correspond to the passive slack sarcomere length.

For most models of the lumbar spine, the kinematics are an input to the model either from subject-specific vertebral motion measurements (Arjmand and Shirazi-Adl, 2006a; Dehghan-Hamani et al., 2019), or as predefined functions that distribute the overall lumbar spine rotation between the moving vertebrae (Christophy et al., 2012; Bruno et al., 2015; Ignasiak et al., 2016). This approach may not be ideal as the spinal forces and moments have been shown to be highly sensitive to input trajectories (Malakoutian et al., 2016b; Eskandari et al., 2019; Dehghan-Hamani et al., 2019; Byrne et al., 2020). For translation specifically, even an error of 0.1 mm is considered too large (Eskandari et al., 2019), whereas such a level of accuracy is not feasible with the current modalities (Malakoutian et al., 2015). Even models that neglect translation and use a predefined, rhythm-based, function for rotation of the vertebrae have been shown to over-predict the joint forces by up to ∼40% (Byrne et al., 2020). In our model, only the rotation (and not the translation) of the thorax was assigned while the other rigid bodies were allowed to move freely with no predefined function. Therefore, the spinal forces/moments in our model were not affected by subjectivity or inaccuracies of the intervertebral input kinematics.

The IDP in this study was calculated as the sum of the IDP resulting from the compressive force and the IDP from the flexion-extension moment both borne by the FSU. Surprisingly, most studies in the literature only relate the compressive forces to IDP and do not consider the IDP from flexion-extension moments (Schultz et al., 1982; Han et al., 2012; Bruno et al., 2015; Senteler et al., 2016; Ignasiak et al., 2016; Bayoglu et al., 2019). Pure flexion and extension moments applied to FSUs increase the IDP such that a 10 Nm moment leads to an IDP of ∼0.36 MPa in flexion and 0.18 MPa in extension (Heuer et al., 2007a; Wilke et al., 2020). Addition of a compressive load to a pure flexion/extension moment has been shown to result in an IDP equal to the summation of the IDPs from each load when applied separately (Schmidt et al., 2007). In our model for 36° flexion, the contribution of IDP from the sagittal plane moment was 36% of total IDP when baseline muscle parameters were chosen but reached 54% when muscle parameters were changed. Therefore, the predictions of those models that ignore the IDP from flexion-extension moments should be reconsidered particularly for activities simulating a flexed posture.

There were a number of limitations in this study. The load sharing between the intervertebral disc and the posterior elements under a compressive force was considered to be 85% for all postures. However, this value has been shown to vary between postures and to be greater in flexion compared to upright standing or extension (Nachemson, 1960; Ghezelbash et al., 2020). The effect of intra-abdominal pressure was modeled as a single force acting on the thorax (normal to the diaphragm surface), which was a simplification. Mechanical stability of the spine was not considered in our solution method. Inclusion of that criterion leads to co-contraction of abdominal muscles, which most optimization models fail to predict (Hajihosseinali et al., 2014). Although inclusion of the stability criterion results in higher forces for upright standing or other light activities, for heavy work activities or postures like flexion where passive structures are more involved, the inclusion of the stability criterion appears not to make a difference (Stokes and Gardner-Morse, 2001; Dreischarf et al., 2016). Adding the stability criterion to our solution method remains a future step. However, even without such a criterion, it is noteworthy that co-contraction of abdominal muscles was evident in our model, especially when passive properties of more muscles (i.e., beyond multifidus) were varied and changed to shorter slack sarcomere lengths or larger stiffness values.

The validation of the model in this study was limited to symmetric tasks and positions within the sagittal plane. In addition, to explore the influence of variation in muscle properties on IDP only two tasks of upright standing and flexing to 36° were simulated. While such analysis on asymmetric tasks would also be of interest, the two tasks considered for this study clearly demonstrated the significant effect of muscle parameters on predicted spinal loading and hence were sufficient for serving the purpose of this study.

The results of this study highlighted the significance of the muscle force-length curve and the parameters associated with it in the prediction of spinal loading; therefore, motivating future models to incorporate those parameters in their model for more accurate results. The results also encourage further experimental studies for measurement of these parameters *in vivo*, especially given the reported wide variations in these parameters.

## Data Availability

The original contributions presented in the study are included in the article/Supplementary Material; further inquiries can be directed to the corresponding author.
