# Supplementary material for: Biomechanical Properties of Paraspinal Muscles Influence Spinal Loading—A Musculoskeletal Simulation Study
Source: Front Bioeng Biotechnol. 2022 Jun 2;10:852201. doi: 10.3389/fbioe.2022.852201 (PMC9201424; doi:10.3389/fbioe.2022.852201)
Supplement: Supplementary file 1 [file DataSheet1.docx]

Supplementary Section

S1- Formulations for Muscle Force Computation

The anatomic properties (typically measured in cadaveric studies) required as input to the musculoskeletal models include the in situ sarcomere length ($l_{cad}^{s}$), PCSA, pennation angle ($\alpha_{cad}$), and the ratio of fiber to musculotendon length ($\frac{l_{cad}^{F}}{l_{cad}^{MT}}$). As these parameters taken from the literature were typically measured in cadavers the subscript “$cad$” was used. Note that absolute values for fiber or tendon lengths are NOT necessary, rather only the ratio of fiber to tendon length is sufficient. Here we explain how in a musculoskeletal model, normalized fiber length $\tilde{l}_{model}^{F}$and consequently the sarcomere length $l_{model}^{S}$ are calculated to be used for computation of muscle forces.

To add muscles to a skeletal model, the attachment sites of each muscle fascicle is identified on bony elements based on detailed description of anatomical studies. By connecting those attachment sites, muscle fascicles are formed. As shown in Figure S1, the resulting length of the connected points (the distance between A and B) is the length of the entire muscle-tendon unit ($l^{MT}$).

The length of the muscle-tendon in the model may not necessarily be the same as the one measured in cadavers; therefore, direct incorporation of cadaveric muscle properties into the model is generally wrong; instead, they should get scaled by the ratio of the muscle-tendon length of the model at t = 0 (before simulation starts) to the muscle-tendon length of the cadaver (i.e. $\frac{l_{model, t=0}^{MT}}{l_{cad}^{MT}}$).

| 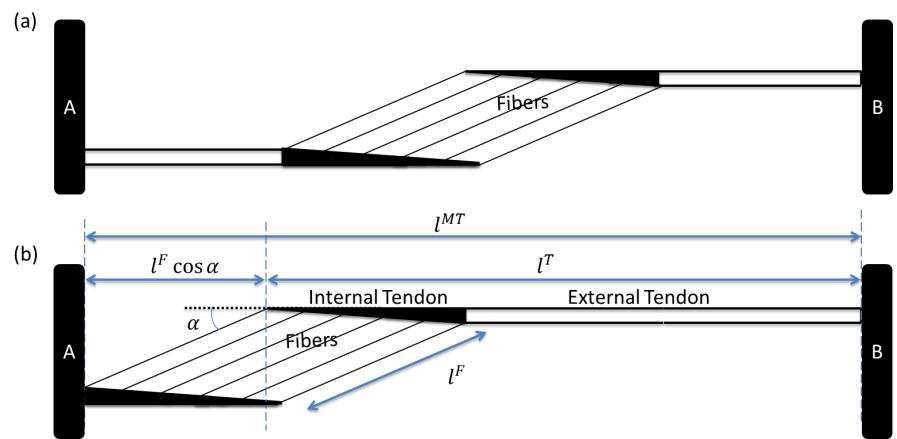 |
| --- |
| Figure S1. Muscle anatomic properties including the pennation angle $\alpha$, musculotendon length $l^{MT}$, and fiber length $l^{F}$. For better demonstration of muscle parameters, (b) is merely a rearrangement of (a). |

With rigid tendon assumption, the length of the model tendon $l_{model}^{T}$ will be constant over time and can be calculated as:

| $l_{model}^{T}=\frac{l_{model, t=0}^{MT}}{l_{cad}^{MT}}\left( l_{cad}^{MT}-l_{cad}^{F}\cos\alpha_{cad} \right)=l_{model, t=0}^{MT}\left( 1-\frac{l_{cad}^{F}}{l_{cad}^{MT}}\cos\alpha_{cad} \right)=cte$ |
| --- |

where $l_{model, t=0}^{MT}$ is the length of the muscle-tendon before the simulation starts (i.e. the body is at rest - supine position just as were the embalmed cadavers in which the muscle properties were measured). To avoid confusion, the parameters in the formulations were color coded: grey represented parameters taken from the literature (typically measured in cadavers), which were all constant values; Blue was used for parameters belonging to the model and were all constant values; and Red was used for model parameters that were dynamic and changed as the body moved.

After tendon length of the model was obtained, the model fiber length $l_{model}^{F}$, which changes as the body moves, can be computed as:

$$l_{model}^{F}=\sqrt{\left( l_{model}^{MT}-l_{model}^{T} \right)^{2}+\left( l_{model, t=0}^{MT}\frac{l_{cad}^{F}}{l_{cad}^{MT}} \sin\alpha_{cad} \right)^{2}}$$

By fiber length changes, the pennation angle changes, the cosine of which could be calculated as:

$$\cos\alpha=\frac{l_{model}^{MT}-l_{model}^{T}}{l_{model}^{F}}$$

Fiber optimum length in the model $l_{o, model}^{F}$ is defined as the fiber length at which sarcomeres are at their optimum length ($l_{o}^{s}$) for generating active forces. This is calculated from the fiber optimum length in the cadaver $l_{o,cad}^{F}$ as:

$$l_{o, model}^{F}=\frac{l_{model, t=0}^{MT}}{l_{cad}^{MT}}\times l_{o,cad}^{F}= \frac{l_{model, t=0}^{MT}}{l_{cad}^{MT}}\times l_{cad}^{F}\times\frac{l_{o}^{S}}{l_{cad}^{S}}= l_{model, t=0}^{MT}\times\frac{l_{cad}^{F}}{l_{cad}^{MT}}\times\frac{l_{o}^{S}}{l_{cad}^{S}}$$

where $l_{o}^{S}$ is assumed for both cadavers and models to be equal to 2.8 *µ*m (Delp et al., 2001); and $l_{cad}^{S}$ is the in situ sarcomere length. Finally, normalized fiber length in the model is computed as:

$$\tilde{l}_{model}^{F}=\frac{l_{model}^{F}}{l_{o, model}^{F}}=\frac{\frac{l_{model}^{F}}{Number of Sarcomeres}}{\frac{l_{o, model}^{F}}{Number of Sarcomeres}}=\frac{l_{model}^{S}}{l_{o}^{S}}=\tilde{l}_{model}^{S}$$

where by “Number of Sarcomeres” it is meant the number of sarcomeres in series that form the length of a muscle fiber; its value is not important here though as it cancels out in the above formula. Therefore, one may obtain the sarcomere length of the model as:

$$l_{model}^{S}=l_{o}^{S}\times\tilde{l}_{model}^{F}$$

The anatomic and biomechanical properties are then used for muscle force computation through the following formulation:

$$F_{muscle}=PCSA\times\left( activation\times SpT\times\tilde{f}_{active}\left( l_{model}^{S} \right)+K\times\tilde{f}_{passive}\left( l_{model}^{S} \right) \right)\times\cos\alpha$$

where $K$ is a constant scaling the normalized passive curve similar to how specific tension (SpT) acts to normalize the active curve; $l_{model}^{s}$ is the model sarcomere length; $\tilde{f}_{active}$ and $\tilde{f}_{passive}$ are force multipliers as functions of $l_{model}^{S}$and are obtained from the normalized force-length curve (Figure 1); and activation is a decimal varying between 0 and 1 representing muscle activation level between turned off and fully activated.

S2 - Calibration

In an in vivo study (Daggfeldt and Thorstensson, 2003) of four male subjects lying on their right side, the maximum voluntary back-extension torques about the L5-S1 joint along with the corresponding intra-abdominal pressures were measured at multiple lying body postures including 20° extension, 10° extension, 10° flexion, and 30° flexion. To simulate this study, gravity was set to zero in our model and an anteriorly oriented horizontal force was applied to T3 center of mass which had a lever arm of 0.39*m* about L5-S1. Division of the measured torques in that study by 0.39 gave us the expected equivalent forces to be applied horizontally at T3 and to be resisted by the model (Table S1). In our model, for each posture the applied horizontal force was increased by increments of 5 N until the model was not able to resist. As a kinematic accuracy with tracking error of less than 1° was desired, the maximum resisting force by the model was defined as the force beyond which the model could not stay within 1° of the prescribed rotation.

A specific tension (SpT) of 100 $N/cm^{2}$ and three weighting terms of 2.5, 0.025, and 0.005 for the first three cost functions were achieved in our previous model through simulation of 10° extension and 10° flexion postures (Malakoutian et al., 2016b). We used these same values and simulated 20° extension and 30° flexion, to determine the weighting term for the fourth cost function ($w_{4}$) which dealt with 6-DOF springs force minimizations and was introduced in this study. Four values of 1, 5, 10, and 15 were attempted for $w_{4}$ to obtain the maximum forces the model could resist to keep the model once within 1° and once within 2° of the prescribed position. With $w4$ = 5 the closest results to the experimental data were achieved by the model, especially for the flexion postures (Table S2).

| Table S1: Average of the maximum sagittal plane moment, intra-abdominal pressure (IAP), and diaphragm area at different positions for subjects of the Daggfeldt et al.’s study (Daggfeldt and Thorstensson, 2003). | | | | |
| --- | --- | --- | --- | --- |
|  | Extension  20° | Extension  10° | Flexion  10° | Flexion  30° |
| Moment Magnitude (*N.m*) | 110 (90-130) | 180 (150-210) | 240 (210-270) | 290 (240-340) |
| IAP (*kPa*) | 12 | 16 | 18 | 18 |
| Diaphragm Area ($cm^{2}$) | 190 (150-230) | 200 (160-240) | 210 (180-240) | 240 (220-260) |
| $F_{IAP}=IAP\times Area$(*N*) | 228 | 320 | 378 | 432 |
| **Expected Equivalent Force to be Resisted by the Model (*N*)** | **282** | **461** | **615** | **743** |

| Table S2: Maximum resistible forces by the model at 20° extension, 10° extension, 10° flexion, and 30° flexion when values of 1,5,10, and 15 were attempted for $w_{4}$ (the weighting term for the FSU forces cost function). Values are reported for tracking errors of 1° and 2°. The weighting term producing closest results to the equivalent forces in Table S1 is presented in bold. | | | | | | | | | |
| --- | --- | --- | --- | --- | --- | --- | --- | --- | --- |
|  | Tracking Error < 1° | | | |  | Tracking Error < 2° | | | |
|  | Extension  20° | Extension  10° | Flexion  10° | Flexion  30° |  | Extension  20° | Extension  10° | Flexion  10° | Flexion  30° |
| w4= 15 | 160 N | 170 N | 205 N | 250 N |  | 225 N | 270 N | 330 N | 400 N |
| w4= 10 | 240 N | 290 N | 350 N | 430 N |  | 410 N | 480 N | 550 N | 615 N |
| **w4= 5** | **400 N** | **505 N** | **615 N** | **745 N** |  | 410 N | 510 N | 650 N | 765 N |
| w4= 1 | 405 N | 555 N | 650 N | 785 N |  | 420 N | 560 N | 700 N | NP |

Once $w4$ = 5 was determined, to refine the value selected for the specific tension, two other values of 80 $N/cm^{2}$ and 90 $N/cm^{2}$were attempted, but still 100 $N/cm^{2}$ was the only one being able to produce sufficient strength especially when the model is flexed (Table S3).

| Table S3: Maximum resistible forces by the model at 20° extension, 10° extension, 10° flexion, and 30° flexion for specific tension. Values are reported for tracking errors of 1° and 2°. The specific tension producing closest results to the equivalent forces in Table S1 is presented in bold. | | | | | | | | | |
| --- | --- | --- | --- | --- | --- | --- | --- | --- | --- |
|  | Tracking Error < 1° | | | |  | Tracking Error < 2° | | | |
|  | Extension  20° | Extension  10° | Flexion  10° | Flexion  30° |  | Extension  20° | Extension  10° | Flexion  10° | Flexion  30° |
| **ST=100** | **400** | **505** | **615** | **745** |  | 405 | 525 | 650 | 765 |
| ST=90 | 375 | 470 | 570 | 695 |  | 385 | 480 | 605 | 695 |
| ST=80 | 345 | 435 | 515 | 640 |  | 360 | 440 | 555 | 640 |
